# Titanium Functionalized with Polylysine Homopolymers: In Vitro Enhancement of Cells Growth

**DOI:** 10.3390/ma14133735

**Published:** 2021-07-03

**Authors:** Maria Contaldo, Alfredo De Rosa, Ludovica Nucci, Andrea Ballini, Davide Malacrinò, Marcella La Noce, Francesco Inchingolo, Edit Xhajanka, Kenan Ferati, Arberesha Bexheti-Ferati, Antonia Feola, Marina Di Domenico

**Affiliations:** 1 Multidisciplinary Department of Medical-Surgical and Dental Specialties, University of Campania Luigi Vanvitelli, Via Luigi de Crecchio, 6, 80138 Naples, Italy; alfredo.derosa@unicampania.it (A.D.R.); ludovica.nucci@unicampania.it (L.N.); 2 Department of Biosciences, Biotechnologies and Biopharmaceutics, Campus Universitario Ernesto Quagliariello, University of Bari “Aldo Moro”, 70125 Bari, Italy; andrea.ballini@uniba.it; 3 Department of Precision Medicine, University of Campania Luigi Vanvitelli, 80138 Naples, Italy; 4 Department of Research, Development and Quality Assessment, AISER SA, Rue du Rhone, 14 VH-1204 Genève, Switzerland; davide.malacrino@aiser.org; 5 Department of Experimental Medicine, Università Degli Studi della Campania Luigi Vanvitelli, Campania, 80138 Naples, Italy; marcella.lanoce@unicampania.it; 6 Department of Interdisciplinary Medicine, University of Medicine Aldo Moro, 70124 Bari, Italy; francesco.inchingolo@uniba.it; 7 Department of Dental Prosthesis, Medical University of Tirana, Rruga e Dibrës, U.M.T., 1001 Tirana, Albania; editxhajanka@yahoo.com; 8 Faculty of Medicine, University of Tetovo, 1220 Tetovo, North Macedonia; kenan.ferati@unite.edu.mk (K.F.); arberesha.ferati@unite.edu.mk (A.B.-F.); 9 Department of Biology, University of Naples “Federico II”, 80138 Naples, Italy; antonia.feola@unina.it; 10 Department of Biology, College of Science and Technology, Temple University, Philadelphia, PA 19122, USA

**Keywords:** cell growth, titanium, polylysine, dental implants, implantology, biomaterials, epithelial growth

## Abstract

In oral implantology, the success and persistence of dental implants over time are guaranteed by the bone formation around the implant fixture and by the integrity of the peri-implant mucosa seal, which adheres to the abutment and becomes a barrier that hinders bacterial penetration and colonization close to the outer parts of the implant. Research is constantly engaged in looking for substances to coat the titanium surface that guarantees the formation and persistence of the peri-implant bone, as well as the integrity of the mucous perimeter surrounding the implant crown. The present study aimed to evaluate in vitro the effects of a titanium surface coated with polylysine homopolymers on the cell growth of dental pulp stem cells and keratinocytes to establish the potential clinical application. The results reported an increase in cell growth for both cellular types cultured with polylysine-coated titanium compared to cultures without titanium and those without coating. These preliminary data suggest the usefulness of polylysine coating not only for enhancing osteoinduction but also to speed the post-surgery mucosal healings, guarantee appropriate peri-implant epithelial seals, and protect the fixture against bacterial penetration, which is responsible for compromising the implant survival.

## 1. Introduction

**Figure 1:**
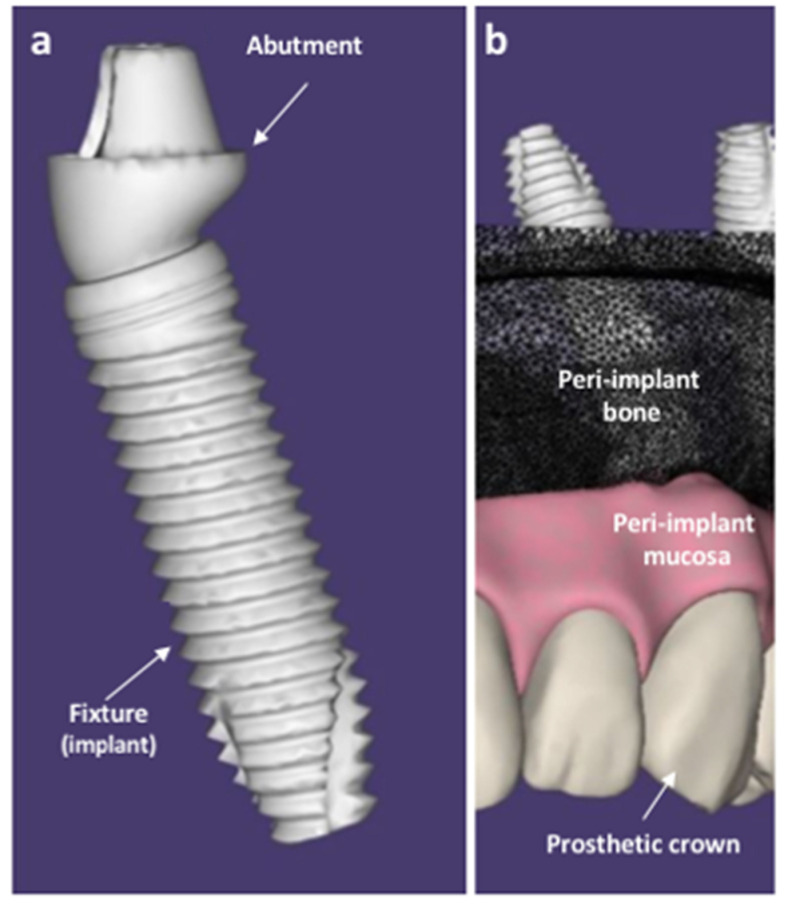
Schematic representation of a dental implant (**a**) and the oral structures (peri-implant mucosa and peri-implant bone) surrounding the fixture (**b**), surgically inserted in the bone. Original figures made by D.M. with SOLIDWORKS ^®^ (CSWP-MBD Version, 2021, SolidWorks, Dassault systems, Waltham, MA, USA).

Dental implants are multi-material prostheses that replace tooth roots with screw-like metal fixtures surgically inserted into the edentulous bone that are connected by the abutment with an artificial crown that replaces the missing tooth, looking and acting identical to the real one (Figure 1).

Dental implants fixtures are generally composed of biomedical titanium and its alloys [1], as they are biocompatible as well as resistant to corrosion and strength [2].

Numerous surgical protocols and variables may affect dental implant placement, and, over the years, novel implantology procedures have been constantly proposed [3,4,5,6].

The main sign of the success of a dental implant is its capability to integrate its shape with the bone and to induct the formation of novel bone around it; these properties are defined as “osseointegration”—“the close contact between the bone and an implant material in histological sections” [7,8]—and “osteoinduction”—the ability to induce the osteogenesis of new mineralized bone around the implant surfaces, thus firmly blocking the fixture within the bones of the jaws [9].

In addition to different surgical protocols [10], geometry modifications [5,11] and various surface treatments for increasing surface roughness [1,12], such as acid-etching, grit-blasting, titanium plasma-spraying, or anodization [13], as well as the use of various coatings to make the titanium surface bioactive [14,15,16,17] are responsible for empowering the wettability, bone anchoring, and biomechanical stability between the implant–bone interfaces [3,6,10,12,18], thus increasing osteoinduction and osteointegration.

Among the coating substances, the polyaminoacid poly-L-lysine has been reported to be able to bridge the cell-adhesion trough covalent attachments to cysteine in the bone [19,20,21].

A model of study on the osteogenic effects of substances is the use of human dental pulp stem cells [22,23,24], which previously has been proven to be involved in bone–implant osseointegration [25,26,27,28]. In details, the role of induced pluripotent stem cells in dentistry has been recently discussed and the use of autologous dental-derived stem cells has been proposed for bone tissue regeneration, as less invasive and more predictable alternative to conventional tissue regenerative procedures [29]. 

Furthermore, the mechanisms underlying the potential effects of poly-L-lysine on these kinds of cells have been reported both in vitro [20] and in vivo on sheep animal models [21].

Bacterial-induced inflammation of the soft tissues surrounding the abutment is the main cause of failure of the osteointegration immediately after the fixture placement and during the years. To avoid bacterial penetration and contamination of the peri-implant bone, which is responsible for inflammation and bone loss, the integrity of the peri-implant seal is crucial [30]. Otherwise, peri-implant inflammation occurs, and the implant survival is compromised. [31,32].

Therefore, a good epithelial attachment between the implant and the peri-implant mucosa is fundamental to achieve and maintain the osteointegration [30,33,34,35], and it is essential to maintain an intact oral epithelial barrier, with no local and systemic risk factors, as bacterial plaque, to offer good resistance to mechanical stress that is both physiological and pathological.

The present work aimed to confirm the in vitro effects of titanium functionalized with a poly-L-lysine coating on human dental pulp stem cells (hDPSCs), which are responsible for osteogenesis, and evaluate analogues effects on keratinocyte cell lines (HaCaT), which are responsible for epithelial attachment of the mucosa surrounding the abutment, to hypothesize a potential improvement of implant osteointegration and the potential use of poly-L-lysine for rapid mucosal healing after the implant placement and during years to preserve the health of peri-implant mucosa.

## 2. Materials and Methods

**Figure 2:**
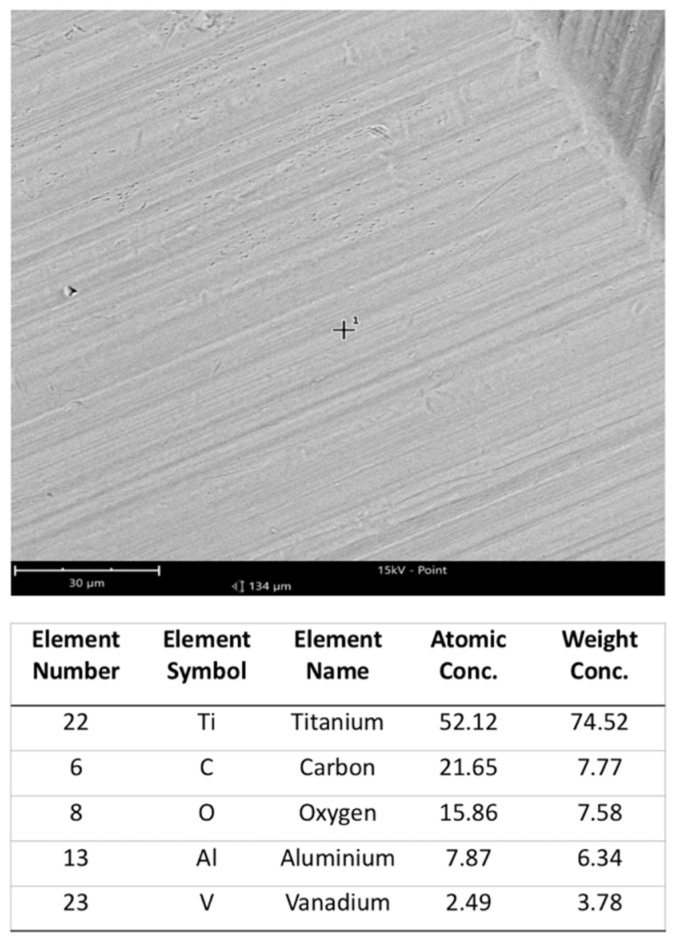
On top, the machined clean titanium plate at SEM. FOV: 134 µm, Mode: 15 kV—Point, Detector: BSD Full. On bottom, the chemical composition analysis of the titanium surface, in spot 1, pointed by a cross in the figure.

**Figure 3:**
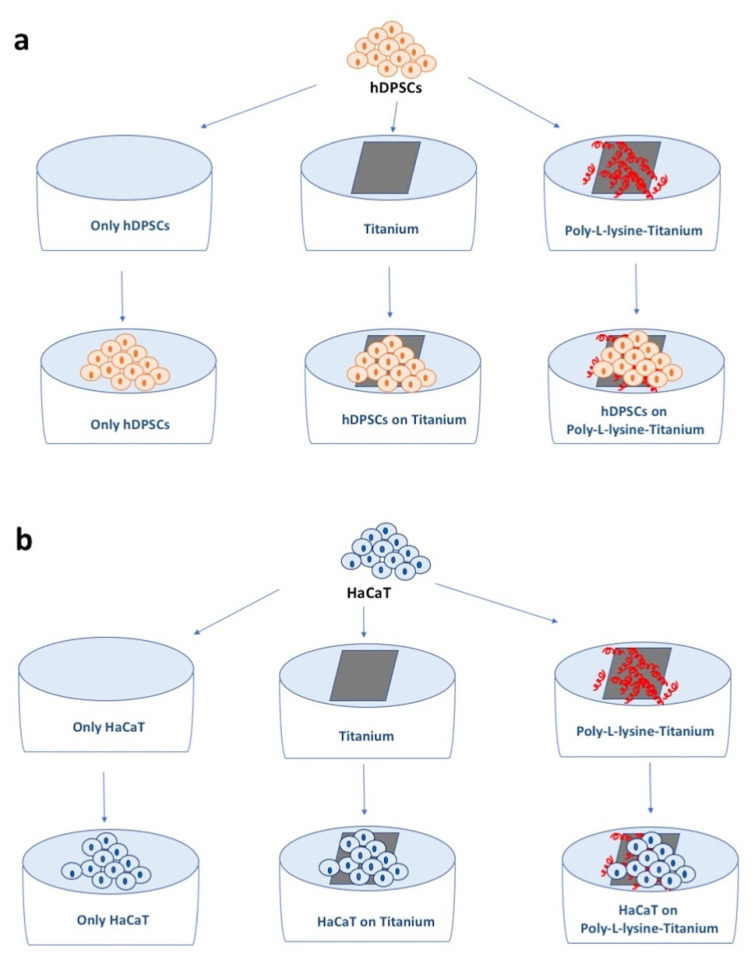
Schematic representation of the experiments. (**a**) hDPSCs cultured alone, on titanium plates, and on titanium plates coated with poly-L-lyisine. (**b**) HaCaT cells cultured alone, on titanium plates, and on titanium plates coated with poly-L-lyisine.

Machined clean square plates (1 cm × 1 cm in size; 0.2 mm thick) made up of 5-Ti-6Al-4V ELI alloy (Klein s.r.l., Milan, Italy) (Figure 2) were sterilized with ethanol 70%, dried under a fume hood, and used in six types of experiments: hDPSCs cultures alone (standard condition), hDPSCs cultures with titanium, and hDPSCs cultures poly-L-lysine-coated titanium (Figure 3a); HaCaT immortalized human keratinocyte line cultures alone, HaCaT cultures with titanium, and HaCaT cultured with poly-L-lysine-coated titanium (Figure 3b). In each experiment, cell viability and proliferation were assessed, as reported below. Sterilized titanium plates were coated with poly-L-lysine incubating at 37 °C for 30 min with a solution containing 0.01% poly-L-lysine and then dried and washed twice with sterile water. After this, cells were cultured on the disks.

### 2.1. hDPSCs Culture and Growth Curve

Experimental procedures were conducted following our previous experience in the field and according to the manufacturer’s specifications [22,23,25,36,37,38,39,40].

Each patient or guardian gave informed consent to tooth extraction obtained with piezo-surgery technology, which was in accordance with the Declaration of Helsinki, for re-use of biospecimens in research applications. Moreover, the study was approved by the Independent Ethical Committee of University Hospital of Bari, Italy (protocol number 155/2021, 27 January 2021). With the purpose to preserve dental tissues for consequent cell isolation and expansion, piezo-surgery technology enables selective tissue cutting, and consequently, tooth buds or embedded third molars can effortlessly be removed from bones with slight wound to periodontal fibers or bud follicles.

In addition, tooth extraction, especially by piezosurgery technique, can be considered less invasive in comparison to bone marrow or other tissues biopsy [22].

Briefly, the pulp was removed and immersed for 1 h at 37 °C in a digestive solution of 3 mg/mL of type I collagenase and 4 mg/mL of dispase in PBS (phosphate buffered saline) containing 40 mg/mL of gentamicin. Once digested, the solution was filtered through 70 μm Falcon strainers (Becton & Dickinson, Franklin Lakes, NJ, USA). Cells were cultured in standard medium consisting of Dulbecco’s modified Eagle’s medium (DMEM) with 100 units/mL of penicillin, 100 mg/mL of streptomycin, and 200 mM l-glutamine (all purchased from Gibco), supplemented with 10% fetal bovine serum (FBS) (Invitrogen, Waltham, MA, USA). Cells were maintained in a humidified atmosphere under 5% CO_2_ at 37 °C, and the media were changed twice a week.

At first passage of culture, cells were seeded at a density of 150.00 cells/titanium implant—with and without poly-L-lysine homopolimers coating—and in standard condition. After 1 h of incubation in 100 μL of culture medium to allow cell attachment, the cell implants and cells cultured without implants were incubated in DMEM at 10% of FBS (fetal bovine serum) into an incubator at 37 °C in a humidified atmosphere consisting of 5% CO_2_ and 95% O_2_ for 24, 48, and 72 h. For each time, an aliquot of cell suspension was diluted with 0.4% trypan blue (Sigma Aldrich, St. Louis, MO, USA), pipetted onto a haemocytometer, and counted under a microscope at 200× magnification. Live cells excluded the dye, whereas dead cells admitted the dye and were consequently stained intensely with trypan blue. The number of viable cells for each experimental condition was counted and represented on a linear graph.

#### MTT Analyses

In order to evaluate the cytotoxicity of titanium implants on cells, MTT assay (3-(4,5-dimethylthiazol-2-yl)-2,5-diphenyltetrazolium bromide) was used [41,42]. Cells, at a density of 300.00 cells/implant with and without poly-L-lysine coating and cells cultured in standard condition (hDPSCs cultured in tissue culture polystyrene (TCP) without titanium and polylysine) were plated in DMEM at 10% FBS for 24, 48, and 72 h. After each time point, the medium was removed, and 200 μL of MTT (Sigma, Milan, Italy) solution (5 mg/mL in DMEM without phenol red) and 1.8 mL of DMEM were added. Four hours later, the formazan precipitate was dissolved in 100 μL dimethyl sulfoxide, and then, the absorbance was measured in an ELISA reader (Thermo Molecular Devices Co., Union City, NJ, USA) at 550 nm. The mean and the standard deviations were obtained from three different experiments of the same specimen.

### 2.2. HaCaT Cells Culture and Growth Curve

HaCaT were cultured in complete culture medium consisting of DMEM (Sigma D5796, Sigma Aldrich, St. Louis, MO, USA) with 1% penicillin/streptomycin (Sigma P0781, Sigma Aldrich, St. Louis, MO, USA), 2 mM glutamine (Sigma G7513, Sigma Aldrich, St. Louis, MO, USA), and supplemented with 10% fetal bovine serum (Sigma F7524, Sigma Aldrich, St. Louis, MO, USA) [43]. All procedures were performed under sterile conditions under a NuAire laminar flow biological hood.

The cultures were expanded in plates every three days in an incubator under 5% CO_2_ at 37 °C (RH = 95%), until the required number of cells was reached. Then, 5 × 10^5^ cells were subsequently transferred to plates containing titanium alone and titanium with poly-L-lysine coating to promote engraftment.

In order to highlight cell clones adhering to titanium, after 48 h, the titanium plates with and without poly-L-lysine coating were removed from the culture and, after suitable washing with PBS twice, they were placed in plates containing only fresh culture medium to observe cell viability.

The cytotoxicity check was performed by culturing the cells in the absence and presence of the titanium plate (with and without poly-L-lysine coating) and by evaluating their viability after replacement in a new fresh medium.

After 72 h, all cells were trypsinized, collected, and evaluated for viability according to tryp blue method using the Burker chamber count (Invitrogen, Milan, Italy).

### 2.3. Statistical Analyses

Student’s test was used for statistical evaluation. A *p*-value < 0.05 was considered significant.

## 3. Results

### 3.1. hDPSCs Growth Curves Analyses

**Figure 4:**
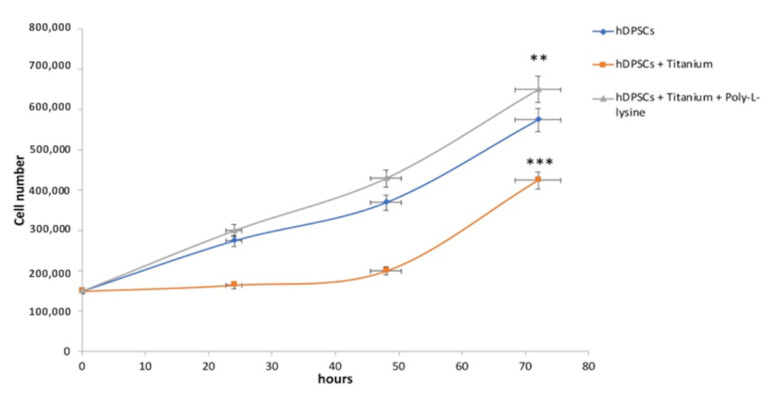
Cell growth analyses. Although hDPSCs cultured in standard condition and on titanium with and without poly-L-lysine showed the same trend in growth, in the culture with titanium coated with poly-L-liysine, the cell growth was higher than the hDPSCs alone and hDPSCs with only titanium. ** *p* < 0.01, *** *p* < 0.001 compared to the hDPSCs.

Cell growth analysis and viability staining with trypan blue showed that hDPSCs cultured in standard condition and on titanium with and without poly-L-lysine showed the same trend in growth; however, while the titanium alone slightly negatively affects the viability for cells (*p* < 0.01), the cell growth on the poly-L-lysine coated titanium was noticeably increased (*p* < 0.001) (Figure 4).

### 3.2. MTT Evaluation in hDPSCs

**Figure 5:**
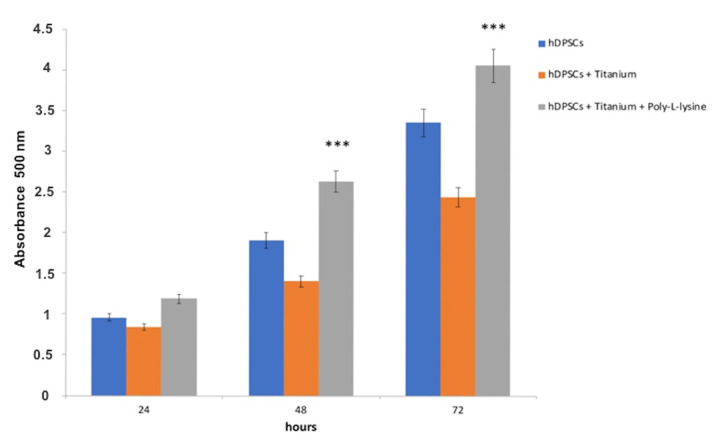
MTT evaluation. Titanium did not show cytotoxicity, and the proliferation of cells seeded on titanium was similar to those of cultured in standard condition, but in titanium coated with poly-L-liysine, cell proliferation was higher at 24, 48, and 72 h. *** *p* < 0.001 compared to the hDPSCs.

To evaluate how the titanium affected the viability and proliferation of hDPSCs, MTT analyses were performed. hDPSCs were cultured on titanium with and without poly-L-lysine coating for 24, 48, and 72 h. Results showed that titanium was not cytotoxic. In addition, there were no changes in terms of proliferation between cells cultured in standard condition and cells seeded on titanium, while the cells seeded on titanium coated with poly-L-lysine showed higher proliferation (*p* < 0.001) (Figure 5).

### 3.3. HaCaT Viability and Proliferation: Mucoproliferative Effects of Titanium

**Figure 6:**
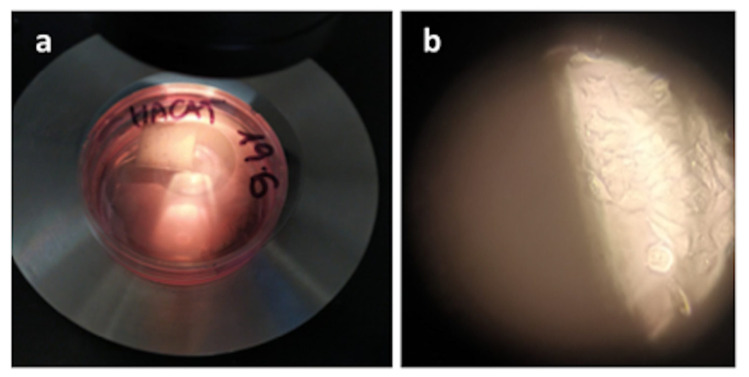
(**a**) HaCat cells culture on the titanium plate coated with poly-L-lysine. (**b**) After a further 48 h, the titanium plate with the adherent HaCaT cells was placed in a new well with a fresh medium, where vital and growing cells were appreciated adhering on the bottom of the well, as shown in figure (**b**) (optical microscopy, original magnification ×10).

To evaluate the viability, HaCaT cells were cultured in the presence of titanium pre-coated with poly-L-lysine. As shown in Figure 6, the mere presence of titanium coated with poly-L-lysine did not affect HaCaT cell viability, as the cells at the bottom of the plate showed normal health morphology [44]. Moreover, cells cultured in the presence of titanium showed an increase in cell proliferation of 40% after 72 h compared to plates containing cells in the absence of titanium. To further demonstrate the presence of live cells adherent on titanium, after 48 h of culture, the titanium plate was placed in a new well with only fresh medium, and even here, adherent keratinocytes were still appreciated. This evidence demonstrates that titanium was found to be a suitable substrate for the viability and growth of keratinocytes in the presence of a culture medium (Figure 6).

## 4. Discussion

The titanium alloys used in dentistry are biocompatible and not cytotoxic, but their surface is also inert, thus not affecting positively the osteoinduction. To empower dental implant osteoinduction, the titanium surface can be functionalized by coating it with a series of bioactive compounds and substances. For this purpose, various coatings have been proposed: nanoparticles of silver, copper, and zinc, as sanitizing agents, and antibacterial and bioactive substances [14], such as quaternary ammonium ions and chlorhexidine, antibiotics, or antimicrobial peptides [15]; calcium-phosphate alone [16] or hydroxyapatite or octacalcium phosphate complexes [17]. These substances are used to make the titanium surface bioactive to improve osteoinduction, by adding, in some cases, antibacterial properties.

Poly-L-lysine is a polyaminoacid carrying positive charges, which increase cellular adhesion on different substrates, and it has been variously reported as an additional coating to titanium surfaces [19,20,21]. In 2005, Spoerke et al. [19] first reported that a nanotextured hybrid titanium coating made up of poly-L-lysine 14% by weight added to calcium phosphate was able to enhance the surface area of the implant and to potentiate the bioactivity of the calcium phosphate alone, by the presence of poly-L-lysine in bridging the cell-adhesion through covalent attachments to cysteine in the bone.

Different studies have reported the osteogenic effects of poly-L-lysine on dental-derived stem cells [22,23,24] and their involvement in bone–implant osteointegration [25,26,27,28]. In 2011, Galli et al. [20] described the potential mechanisms by which poly-L-lysine can enhance osteogenesis, thus reporting that hMSCs (human mesenchymal stem cells) and hDPSCs cultured on poly-L-lysine-treated titanium (Ti6Al4V) showed significantly higher expression of bone marker genes, produced a higher quantity of calcium deposits, and showed higher cell viability after 12 h of culture in comparison with the cells on the untreated titanium. These effects were allowed both by the poly-L-lysine positive charges and its interaction with β-integrin and other molecules from the extracellular matrix (as collagen I, fibronectin, and vitronectin) and their adhesion receptors on the studied cells, thus activating the intracellular signaling cascade responsible for the upregulation of osteogenic markers genes. Among the osteogenic markers activated, alkaline phosphatase is responsible for focal adhesion kinase (FAK) phosphorylation. While the unphosphorylated FAK is capable of blocking the mineral deposition, conversely, phosphorylated FAK (p-FAK) increased in the presence of titanium treated with poly-L-lysine and promoted calcium deposition, osteogenic differentiation, and bone maturation. In support of this mechanism, the same authors reported the presence of p-FAK only in cells treated with titanium-poly-L-lysine and from a twofold (at day 6) to eightfold (at day 25) increase of osteogenic differentiation markers in hMSCs and hDPSCs grown on titanium and poly-L-lysine compared to untreated hMSCs and hDPSCs [20]. In conclusion, poly-L-lysine seems to increase the p-FAK form, thus limiting its capability to block the mineral deposition and hence promoting the osteoblastic differentiation pathway and initiating mitogen-activated protein kinases, leading to osteogenic differentiation and bone maturation.

Four years later, in 2015, Varoni et al. confirmed the effect of poly-L-lysine coatings on titanium osseointegration by in vivo studies on sheep animal models [21]. Their results showed that cortical bone microhardness significantly improved in the presence of the poly-L-lysine coating by enhancing calcium deposition and implant early osseointegration in animals.

Little literature exists about the proliferative effects of poly-L-lysine on HaCaT cells; the work closest to highlighting this effect was the study by Renò et al. [45], but a complete and exhaustive explanation of the underlying mechanisms has not been reported yet. Renò et al. tested the efficacy of two different hydrogels synthesized by crosslinking gelatin with polylysine (positively charged) (HG1) and gelatin with polyglutamic acid (negatively charged) (HG2) as scaffolds for immortalized human keratinocytes (HaCaT) growth. They found that keratinocytes adhered both onto the HG1 and HG2 surface and were capable of proliferating, without toxicity, even if the cells displayed higher adhesion and proliferation onto HG2, forming a continuous and stratified epithelium after 7 days [45]. Further studies are necessary to elucidate the poly-L-lysine effects on epithelial cells and wound-healing processes in depth.

To prevent bacterial infections and facilitate the bone mineralization around the dental implants, recently, Guo et al. reported the synergistic effect of a composite coating made up of poly-L-lysine/sodium alginate and nanosilver [46], while Zhang et al. coated the titanium surfaces with a multilayer biofilm of ε-polylysine and arabic gum [47].

The present work tested the effects of poly-L-lysine-coated implant plates on the cell growth and cytotoxicity both on epithelial cells and dental-derived stem cells, in order (i) to confirm any proliferative effects on mesenchymal cells responsible for osteogenesis and (ii) to establish whether it exerts a potential similar muco-proliferative effect on cells of epithelial origin. For these purposes, a series of experiments were conducted on two different cell lines: epithelial (HaCaT) and mesenchymal (hDPSCs) cells.

Results unanimously have reported cell viability, lack of cytotoxicity, and a statistically significant improvement of the cell growth both for hDPSCs and HaCaT when cultured on poly-L-lysine-coated titanium plates, when compared with the cultures of the cells alone and those of the cells with uncoated titanium plates.

## 5. Conclusions

The oral cavity is always challenged by mechanical, chemical, and biological stimulations throughout life [48], and because the oral mucosa represents a protective barrier between the soft tissues and the external environment [49], it is essential to preserve its integrity and resistance to mechanical stress, both physiological and pathological, and to reduce irritating local factors such as bacterial plaque [30,50]. Oral dysbiosis and poor oral hygiene compromise the health of the peri-implant soft tissues. Furthermore, as in gingivitis and periodontitis, which are diseases responsible for gingival inflammation and bone loss strictly associated with bacterial plaque composition and bone diseases such as osteoporosis [51,52], peri-implant sites can be equally affected by their counterparts as well. These counterparts are called mucositis and peri-implantitis [31,32] which, respectively, lead to inflammation of the mucosa surrounding the abutment and the loss of bone around the fixture, thus compromising the stability of the implant in the bone, which is resorbed and decreased [53,54,55,56]. Furthermore, dysmetabolic diseases such as chronic hyperglycemia have been associated with periodontitis and peri-implantitis due to delayed and/or impaired wound healing for the activation of pathways linked to inflammation, oxidative stress, and cell apoptosis [57,58,59].

The present work is an exploratory study to confirm the bone proliferative effects of a poly-L-lysine coating on titanium and to establish analogue proliferative effects on keratinocytes and lack of cytotoxicity.

The results have confirmed the positive effects of poly-L-lysine on osteoinduction [20,21,28,42,53] and demonstrated a novel potential role also in promoting epithelial cell growth. It means that in clinical practice, a poly-L-lysine topic administration on the surgical mucosal site of a dental implant, could promote, accelerate, and ameliorate the formation of epithelial tissue around semi-submerged and on submerged implants, to favor more rapid healing of the surgical site after the fixture placement and to reinforce the epithelium surrounding the abutment during the remaining life of the implant, thus preventing mucositis and peri-implantitis arising from a loose gingival–implant thigh contact.

However, further in vivo studies are required to confirm the effects of titanium functionalized with a poly-L-lysine coating to improve implant osteointegration and to elucidate the mechanisms of action on keratinocytes and the in vivo efficacy of polylysine compounds in promoting epithelial cell growth and wound healing, as well as after the implant placement and during years to preserve the health of peri-implant mucosa, with particular attention to the aesthetic area [60].

Furthermore, the additional in vivo studies could be supported by non-invasive imaging techniques [61,62,63,64,65] as well as classical procedures, which could highlight and quantify the real histological and cytologic effects of poly-L-lysine on epithelial cell growth to enhance and/or support the wound healing not only at peri-implant sites but also for the treatment of oral lesions and injuries requiring the re-establishment of a healthy mucosal barrier [66,67,68,69] and the reduction of biofilm formation around the teeth and implants [70].

## Data Availability

Data sharing is not applicable to this article.
